# Metabolism meets transport: a new perspective on auxin homeostasis at subcellular level

**DOI:** 10.1007/s00425-026-05083-y

**Published:** 2026-08-19

**Authors:** Ludmila Včelařová, Jakub Lemberk, Ondřej Novák, Aleš Pěnčík

**Affiliations:** 1 Laboratory of Growth Regulators, Faculty of Science, Palacký University, Šlechtitelů 27, 77900 Olomouc, Czech Republic; 2 Laboratory of Growth Regulators, Institute of Experimental Botany of the Czech Academy of Sciences, Šlechtitelů 27, 77900 Olomouc, Czech Republic

**Keywords:** Auxin, Metabolism, Conjugation, Homeostasis, Cell compartments, Auxin transport

## Abstract

**Main conclusion:**

**This review highlights recent advances revealing auxin homeostasis as an integrated network of metabolism and transport, emphasizing subcellular compartmentalization as a key regulatory principle.**

**Abstract:**

Indole-3-acetic acid (IAA), the principal auxin, is a central regulator of plant growth and development throughout the plant life cycle. Recent discoveries have reshaped our understanding of auxin metabolism and highlighted its close integration with auxin transport in maintaining cellular homeostasis. This review summarizes current knowledge on IAA metabolism and places it into the broader framework of auxin distribution within plant cells. The spatial distribution of auxin is tightly linked to its metabolic regulation, as localized auxin accumulation underlies many developmental and signaling processes. Subcellular compartmentalization of auxin into organelles such as the endoplasmic reticulum or vacuole is closely associated with metabolic modifications that influence auxin transport and availability, although the mechanisms governing the movement of auxin conjugates across organellar membranes remain poorly understood. Directed auxin transport delivers active IAA to specific intracellular sites where enzymes catalyze its conjugation, hydrolysis, or degradation. Together, these interconnected processes create a dynamic regulatory network that coordinates auxin metabolism, transport, and compartmentalization to maintain auxin homeostasis and ensure precise control of plant growth and development.

## Introduction

Plant hormones are unique naturally occurring low-molecular-weight substances that, even at low concentrations, significantly influence plant physiological processes including growth, differentiation, and responses to biotic and abiotic stress (Williams 2021). The first studied phytohormones were auxins, whose discoverers are considered to be Charles Darwin and his son Francis Darwin, who showed that light and gravity are perceived by the tips of roots and shoots and that they respond to asymmetric stimulation by curving, although the role of the root apex in gravity perception had already been experimentally demonstrated earlier by Theophil Ciesielski (Ciesielski 1872; Darwin and Darwin 1880).The term auxin, derived from the Greek “auxien” meaning "to grow", was first used by Fritz Kögl and Arie Jan Haagen-Smit (Kögl and Haagen-Smit 1931). Auxin, specifically indole-3-acetic acid, was surprisingly isolated for the first time from human urine (Kögl et al. 1934b), from yeast (Kögl et al. 1934a) and subsequently also from mold *Rhizopus suinus* (Thimann 1935). In vascular plants, IAA was identified only in 1946 in immature *Zea mays* kernels (Haagen-Smit et al. 1946). Since then, the occurrence of IAA and other naturally occurring auxins has been confirmed and studied in a wide range of vascular plant species (Hladík et al. 2023; Matsuda et al. 2005; Quittenden et al. 2009), bacteria (Přikryl et al. 1985; Pintado et al. 2023), fungi (Basse et al. 1996; Chen et al. 2023; Tanaka et al. 2011), moss (Ashton et al. 1985; Drábková et al. 2015) and algae (Ashen et al. 1999; Cheng et al. 2023; Stirk et al. 2013). IAA is not only the most important auxin but also one of the most extensively studied plant hormones playing a central role in regulating plant growth.

Auxins are involved in all aspects of plant development and reaction to the environment. Typical processes strongly regulated by auxin include embryogenesis, organogenesis, responses to light and gravity, and lateral and adventitious root formation (Cavallari et al. 2021; Konstantinova et al. 2021; Roychoudhry and Kepinski 2022). The distribution and levels of IAA within tissues, cells, or even organelles are tightly regulated through precise control of its biosynthesis, metabolism, and transport.

IAA is primarily synthesized through the indole-3-pyruvic acid (IPyA/IPA) pathway, which is considered the most prominent among the tryptophan-dependent auxin biosynthesis routes (Eklund et al. 2015; Mashiguchi et al. 2011; Stepanova et al. 2008; Tao et al. 2008; Tivendale et al. 2012). This pathway involves two key enzymatic steps: the conversion of tryptophan to IPyA by TRYPTOPHAN AMINOTRANSFERASE OF ARABIDOPSIS (TAA) and its homologs, followed by the decarboxylation of IPyA to IAA by flavin-containing monooxygenases from the YUCCA family (Mashiguchi et al. 2011; Stepanova et al. 2011; Zheng et al. 2013). YUCCA flavin monooxygenase–like enzymes (YUC) were first identified as key regulators of auxin biosynthesis when overexpression of YUC genes was shown to induce auxin overproduction phenotypes in Arabidopsis (Zhao et al., 2001). Although this discovery established YUC proteins as essential components of the auxin biosynthetic pathway, their precise biochemical role was not initially resolved, and alternative intermediates, including N-hydroxytryptamine, were proposed. Subsequent genetic and biochemical analyses reassessed these early models and helped clarify the enzymatic function of YUC proteins (Tivendale et al., 2010). The structure of the transmembrane domain and the subcellular location of YUC proteins are thought to be linked to their specific roles and distribution in tissues. YUC4 in *Arabidopsis* has two major isoforms, YUC4.1 and YUC4.2, with YUC4.2 specifically localized to the cytosolic side of the endoplasmic reticulum (ER) membrane (Kriechbaumer 2016). This isoform is active only in flowers, highlighting its specialized role in floral development (Cheng and Zhao 2007). Phylogenetic analysis suggests that ER localization of YUC proteins originated early in moss evolution (Poulet and Kriechbaumer 2017). The IPyA pathway is evolutionarily conserved across various plant species, from mosses to flowering plants, underscoring its fundamental role in plant development (Carrillo-Carrasco et al. 2023; Eklund et al. 2015).

Although the IPyA pathway clearly represents the central route of IAA biosynthesis, several additional TRP-dependent pathways have been proposed (Tivendale et al. 2014). However, their physiological relevance and contributions to overall auxin production remain far less understood. Recent work by Fenech et al. (2025) provides a comprehensive reassessment of auxin biosynthetic pathways in Arabidopsis. The results confirmed that the IPyA pathway is the main and best-supported route for the biosynthesis of IAA. The study has also validated that exogenously supplied precursors such as indole-3-acetonitrile (IAN) and indole-3-acetamide (IAM) can be converted into IAA in vivo. This conversion is mediated by NITRILASE enzymes (NIT1–NIT4) for IAN and IAM HYDROLASE enzymes (IAMH1 and IAMH2) for IAM. However, these conversions do not occur as part of a unified, direct endogenous pathway. In contrast, the previously proposed linear indole-3-acetaldoxime (IAOx) pathway, where IAOx is sequentially converted to IAN, then to IAM, and finally to IAA, is disproved. Knockout mutants of the entire different IAA biosynthetic gene families showed no auxin-deficient phenotypes, did not suppress the high IAA levels in the overproducing mutant superroot 2 (sur2), and exhibited no significant changes in IAA-related metabolic profiles. These findings indicate that previously proposed biosynthetic genes are not required for converting IAOx to IAA in vivo.

While a tryptophan-independent IAA biosynthesis pathway was hypothesized based on studies in Arabidopsis and *Zea mays* (Normanly et al. 1993; Wright et al. 2001; Wang et al. 2015), more recent findings suggest that earlier results supporting this pathway might be explained by the tryptophan-dependent route (Nonhebel 2015). Nevertheless, tryptophan-independent pathways have been confirmed in certain microorganisms, where indole or indole-3-glycerol phosphate serve as precursors for IAA synthesis (Spaepen et al. 2007). Advances in research continue to refine our understanding of these pathways, emphasizing the complexity and versatility of auxin biosynthesis in plants.

In addition to de novo biosynthesis, IAA can be released from low- or high-molecular-weight storage conjugates with the involvement of specific hydrolases (Ludwig-Müller 2011), or from chain-lengthened precursor indole-3-butyric acid (IBA) (Bartel et al. 2001). IBA is converted to IAA via a fatty acid β-oxidation pathway associated with the peroxisome, catalyzed by several enzymes, including dehydrogenase/reductase IBR1, acyl-CoA dehydrogenase/oxidase-like IBR3, enoyl-CoA hydratase2 (ECH2), and predicted enoyl-CoA hydratase (IBR10), with mutants in these enzymes exhibiting defects in apical hook curvature, lateral root formation, and free IAA levels (Zolman et al. 2007, 2008; Strader et al. 2010, 2011). Furthermore, impaired conversion of isotopically labelled IBA-to-IAA in peroxisome-defective mutants provides strong experimental evidence for a central role of the peroxisome in IBA metabolism and IBA-to-IAA conversion (Strader et al. 2010). More recently, LONG CHAIN ACYL-CoA SYNTHETASE 4 (LACS4) was identified as an additional component of this pathway, catalyzing the activation of IBA prior to its β-oxidative conversion in the peroxisome (Jawahir and Zolman 2021).

Early studies reported conversion of IAA to IBA in maize seedlings and in enzymatic preparations from maize and Arabidopsis (Ludwig-Müller and Epstein 1992; Ludwig-Müller and Hilgenberg 1995; Ludwig-Müller 2007). However, no genes, enzymes, or mutants specifically involved in this process have been conclusively identified, and definitive evidence for physiologically relevant IAA to IBA conversion in planta remains lacking, so the concept of reversible IBA-IAA interconversion remains hypothetical. Identifying the enzymes responsible for IBA biosynthesis remains a major challenge, as mutants defective in IBA synthesis will provide important tools for elucidating the biological functions of IBA in plants (Frick and Strader 2018).

## Reversible auxin inactivation

Conjugation of IAA to various molecules serves as a key mechanism for its temporary storage and modulation, while hydrolysis of these conjugates enables the reactivation of free IAA when needed. Understanding these dynamic processes provides insight into auxin homeostasis and its role in plant adaptive responses.

IAA can be reversibly inactivated through amide linkage with amino acids, peptides and proteins or through ester linkage with sugars or through methylation (Korasick et al. 2013). Over the years, a number of conjugates have been identified in plants. The most common amide-linked conjugates found across various plant species are those formed with amino acids aspartate (IAA-Asp), and glutamate (IAA-Glu) (Novák et al. 2012; Matsuda et al. 2005; Široká et al., 2022; Hladík et al. 2023). Other IAA conjugates, including those with alanine, glycine, leucine, phenylalanine, and valine, have also been reported in plants, though they occur in significantly lower quantities (Kowalczyk and Sandberg 2001; Pěnčík et al. 2009; Staswick 2009).

The formation of amide conjugates is catalyzed by the IAA amidosynthase enzymes of the GRETCHEN HAGEN (GH3) family, which were first identified in soybean (Hagen et al. 1984). Since then, GH3 genes have been identified and characterized in numerous plant species, includidng *Arabidopsis* (Staswick et al. 2005), *Zea mays* (Feng et al. 2015) or *Physcomitrium patens* (Ludwig-Müller et al. 2009). So far, 19 genes encoding GH3 enzymes have been identified in *Arabidopsis*, which have been phylogenetically divided into three groups (I-III) based on their structure and functional characteristics, whereby members of group II function as IAA-amide synthetases (Staswick et al. 2005). Increased auxin concentrations lead to strong induction of GH3 enzymes (Chen et al. 2010). These enzymes contribute to a negative feedback loop controlling IAA levels (Hayashi et al. 2021). IAA amide conjugates have already been shown to have a storage function. However, research in recent years suggests that they have a signaling effect in various biological processes. For example, the effect of the dominant amide conjugate IAA-Asp on abiotic stress, specifically metal stress and salinity stress, has been described in pea (Ostrowski et al. 2016).

In *P. patens*, two GH3 enzymes, designated PpGH3-1 and PpGH3-2, have been identified and functionally characterized (Bierfreund et al. 2004). PpGH3-1 exhibits a substrate preference for alanine and asparagine, whereas PpGH3-2 demonstrates broader specificity, conjugating IAA with amino acids such as aspartate, glutamate, tryptophan, and valine. Functional analyses of knockout mutants indicate that these enzymes are essential for maintaining auxin equilibrium and play a significant role in stress responses, particularly under conditions of elevated salinity (Koochak and Ludwig-Müller 2021). Phylogenetic studies show that *Picea abies* possesses multiple GH3 genes. Expression analysis indicates that nine Group II *PaGH3* genes are upregulated after IAA treatment, similar to angiosperms. Substrate specificity highlights functional divergence within the GH3 family while preserving their role in auxin homeostasis. PaGH3.17 prefers glutamate, whereas PaGH3.16, PaGH3.gII.9, and PaGH3.gII.8 favor aspartate, suggesting distinct contributions to auxin regulation in spruce (Brunoni et al. 2020). This specialization may reflect adaptations to the physiological and developmental needs of conifers, potentially influencing auxin turnover, storage, and response to environmental cues.

Hydrolysis of amide conjugates leading to a release of active IAA is catalyzed via amidohydrolases of the IAA-LEUCINE RESISTANT 1/ILR1-LIKE PROTEINS (ILR/ILL) family (Fu et al. 2019). Seven amidohydrolases have been described in *Arabidopsis*– ILR1, ILL1, ILL2, ILL3, IAR3 (ILL4), ILL5, and ILL6. Enzymes ILR1, ILL1, ILL2, and IAR3 have been the best characterized enzymes so far due to in vitro enzymatic assays (LeClere et al. 2002). The results of the enzymatic assays together with auxin metabolic analysis of a range of mutants then confirmed the specificity of the hydrolases toward specific auxin amide conjugates. IAR3 and ILL2 showed high catalytic activity toward the substrate IAA-Ala, while ILL1 preferred IAA-Phe and IAA-Leu (LeClere et al. 2002). For IAR3, the specificity was later extended to IAA-Leu (Sanchez Carranza et al. 2016). Although IAA-Asp and IAA-Glu are among the most abundant auxin conjugates, they were long considered irreversible due to a lack of evidence for their hydrolysis back to free IAA (Ludwig-Müller 2011).

Only with advanced biochemical and genetic methods did evidence emerge challenging this classification, indicating that some enzymes might partially hydrolyze these conjugates (Hayashi et al. 2021; Casanova-Sáez et al. 2022). This shift in perspective opens new questions about the dynamics of auxin metabolism and the possible involvement of these conjugates in the regulatory mechanisms of auxin signaling.

IAR3, ILL2, and ILR1 have been shown to localize to the ER using translational fusions with the fluorescent protein (Carranza et al., 2016). ILR1 homologs are relatively abundant in plants outside Arabidopsis. For example, ILR1-like SlILL homologs have been identified in *Solanum lycopersicum* (Fu et al. 2019), *Triticum aestivum* (Campanella et al. 2004), and *Brassica rapa* (Savić et al. 2009). Moss lacks ILL enzymes, and liverworts like *Marchantia* do not use ILR1 for auxin conjugate hydrolysis (Campanella et al. 2018; Brunoni et al. 2020). This points to unidentified enzymes driving auxin conjugate hydrolysis in non-vascular land plants (Brunoni et al. 2023).

Ester conjugates are formed by attaching a sugar moiety to a carboxyl group via an ester bond. The most prominent ester-linked auxin conjugate IAA-glucose (IAA-glc) has been determined in various plant species over the years, including *Arabidopsis* (Tam et al. 2000; Včelařová et al. 2021), rice (Ishimaru et al. 2013) or spruce (Brunoni et al. 2020) and it is assumed to be important storage form of auxin primarily accumulated in the vacuole (Ranocha et al. 2013). Another auxin ester conjugate IAA-*myo*-inositol (IAInos) has also been identified in several plant species (Bandurski et al. 1969; Fu et al. 2019). In maize, formation of IAInos plays significant role in controlling auxin concentration during seedling development and influences the response of maize to drought and cold (Ciarkowska et al. 2022). The formation of auxin esters is catalyzed by uridine diphosphate (UDP) glucosyl-transferase enzyme family (UGTs) (Jackson et al. 2001; Jin et al. 2013; Szerszen et al. 1994). As an alternative to ester-linked glucosides, the sugar moiety can be attached to IAA via the nitrogen atom of the indole ring, forming *N-*glucosides. The *N-*glucoside of IAA (IAA-*N*-Glc) has been found in several species is predominantly accumulated in seeds (Ljung et al. 2001; Kai et al. 2007). To date, the only known hydrolase capable of releasing IAA from glucose conjugates is THOUSAND-GRAIN WEIGHT 6 (TGW6) identified in rice (Ishimaru et al. 2013).

## Oxidative catabolism and decarboxylation

The oxidative catabolism of IAA represents a crucial pathway for the irreversible degradation of auxin, contributing to the fine-tuned regulation of its homeostasis. As the first product of IAA oxidative catabolism, 2-oxindole-3-acetic acid (oxIAA) was identified in *Zea mays* (Reinecke and Bandurski 1981). Later, oxIAA was also determined in *Arabidopsis thaliana* (Östin et al. 1998; Kowalczyk and Sandberg 2001) and a number of other plant species. OxIAA has been demonstrated to be an irreversible IAA catabolite without auxinic activity, and its formation through IAA oxidation serves a key mechanism regulating auxin homeostasis, influencing its gradients and maxima/minima in the root apex (Pěnčík et al. 2013).

Despite early identification, the biochemical mechanism of IAA oxidation and the enzymes involved have remained largely unknown, making it challenging to determine the impact of IAA catabolism in plants. This changed with the identification of *DIOXYGENASE FOR AUXIN OXIDATION (DAO)* in rice (Zhao et al., 2013) and its two homologs *DAO1* and *DAO2* in Arabidopsis. The DAO1 enzyme catalyzes the time- and tissue-specific oxidative inactivation of IAA (Porco et al., 2016). The *DAO1* gene is expressed in cells of the distal elongation zone and root maturation zone, root columella, and atrichoblasts, whereas *DAO2* is weakly expressed in the root columella and procambium of the root maturation zone (Zhang et al. 2016). OxIAA can be conjugated with glucose by the enzyme UGT74D1 to oxIAA-glc (Tanaka et al. 2014; Mateo-Bonmatí et al. 2021), which represents the predominant low-molecular-weight conjugate in *Arabidopsis* (Kai et al., 2007a; Hladík et al. 2023). Additionally, the presence of oxIAA-amino acids in plants was demonstrated in various species (Kai et al., 2007a; Hladík et al. 2023).

OxIAA may be further oxidized to 7-OH-OxIAA in maize (Lewer and Bandurski 1987) or to 3-hydroxy-oxIAA (dioxIAA), which is predominantly present as its amino acid conjugates (Tsurumi and Wada 1986; Isobe and Miyagawa 2022). However, the mechanism of formation of these oxidation products has not yet been described.

In addition to the well-characterized oxidation route, recent evidence demonstrates that IAA also undergoes decarboxylative degradation. Using stable-isotope tracing and non-targeted LC–MS analysis in tobacco BY-2 cell culture, Dobrev et al. (2023) identified indole-3-carbinol (I3C) and oxindole-3-carbinol (OxI3C) as the first intermediates of these pathways, formed predominantly in the extracellular space by peroxidase activity. These metabolites and their downstream conjugates, including ascorbigen, dihydro-ascorbigen, and glutathione adducts, were subsequently confirmed to occur in planta across multiple species, indicating that the decarboxylative route is not a BY-2-specific phenomenon. Notably, in *Arabidopsis thaliana* several of these compounds also arise from glucosinolate degradation, highlighting a biochemical intersection between auxin catabolism and secondary metabolism. The demonstration that decarboxylative IAA metabolism operates in living tissues revives the longstanding idea that some physiological auxin responses may be mediated by reactive decarboxylative products rather than IAA itself.

## GH3-ILR1-DAO pathway

**Fig. 1:**
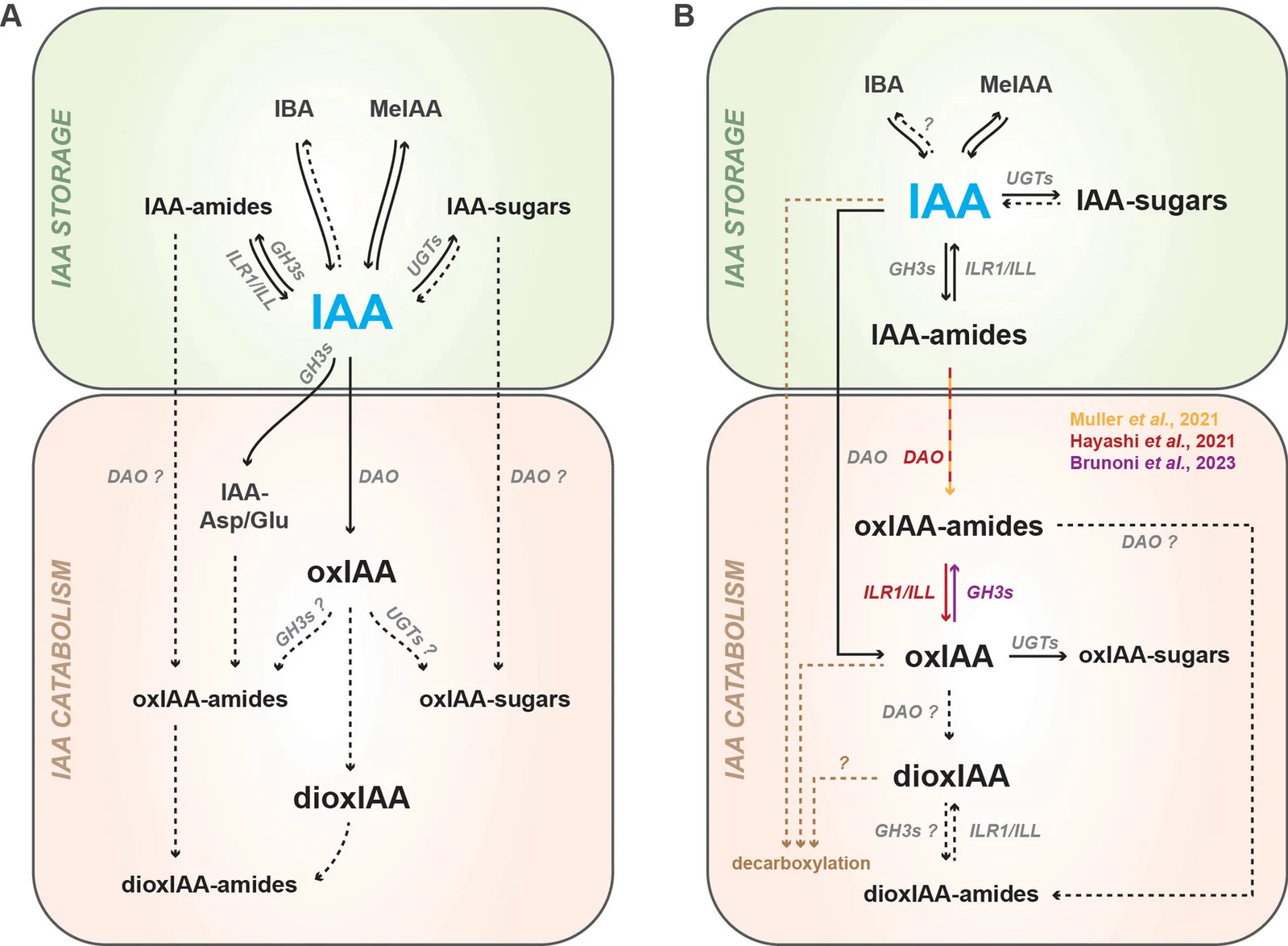
Conventional (**A**) and updated (**B**) model of major auxin inactivation pathways (Zhao et al. 2013; Porco et al., 2016; Müller et al. 2021; Hayashi et al. 2021; Brunoni et al. 2023)

The original model of IAA metabolism, recognized for several decades, describes conjugation to the amino acids via GH3s and oxidation by DAO as two parallel, independent pathways of IAA inactivation (Fig. 1A). Porco et al. (2016) demonstrated that the inactivation of *DAO1* leads to a significant accumulation of IAA-Asp and IAA-Glu, which was interpreted as a compensatory mechanism ensuring the maintenance of auxin homeostasis. However, the identification of oxIAA amino acid conjugates suggested the existence of an interconnecting pathway between these two processes, though evidence regarding their role and formation remained elusive. In recent years, the understanding of auxin inactivation pathways has dramatically changed with increasing experimental support.

The study of Müller et al. (2021) focused on auxin metabolism in auxin-dependent tobacco BY-2 cells, revealing that auxin starvation leads to a significant decrease in oxIAA-Asp levels, accompanied by the downregulation of DAO1 homologs at both transcript and protein levels. Functional analysis in NtDAO1 mutants and Arabidopsis *dao1-1* plants, along with an enzyme assay, confirmed that DAO1 directly oxidizes IAA-amino acid conjugates. A complete shift in the understanding of IAA metabolic pathways was brought by Hayashi et al. (2021), who demonstrated that in Arabidopsis, oxIAA is primarily produced through the hydrolysis of oxIAA amino conjugates, specially oxIAA-Asp and oxIAA-Glu, catalyzed by ILR1/ILL hydrolases, rather than through the direct oxidation of IAA. In the newly proposed model, conjugation and oxidation are not parallel but sequentially interconnected mechanisms. Moreover, the authors provided evidence that IAA-Asp and IAA-Glu serve as storage forms of IAA and can be converted back into active IAA by ILR1/ILL hydrolases (Fig. 1B).

According to the new model, dioxIAA appears as a downstream degradation product that originates from oxIAA-Asp and oxIAA-Glu. However, it has not yet been confirmed whether it is formed exclusively through the oxidation of oxIAA or if an alternative pathway bypassing oxIAA via dioxIAA conjugates is involved. Additionally, the enzymes responsible for dioxIAA synthesis and its conjugates have yet to be identified, making this a key focus of future research.

Studies on auxin metabolism in non-flowering plants indicate that the proposed model is not universal and that auxin homeostasis pathways are species-dependent. In spruce, DAO-like enzymes do not lead to the oxidative inactivation of IAA or its conjugates, suggesting that GH3-mediated conjugation serves as the primary auxin inactivation pathway in conifers (Brunoni et al. 2020). Further investigations suggest that the GH3-ILR1-DAO pathway likely does not operate in early land plants, whereas GH3-mediated formation of oxIAA-amides represents a minor yet functional auxin inactivation route in *P. patens* as well as in Arabidopsis (Brunoni et al. 2023).

## (Sub)cellular auxin gradients and homeostasis

Although the biosynthesis, metabolism, and spatial distribution of IAA in plants have been studied for many years, the precise mechanisms underlying the maintenance of auxin homeostasis at the intracellular level remain elusive. Compartmentalization is widely recognized as a fundamental characteristic of eukaryotic cells. This structural organization creates specific microenvironments and conditions that support specialized metabolic processes, with each organelle housing a unique set of enzymes, transporters, and other proteins.

Auxin signaling has traditionally been understood as a nuclear, transcription-dependent process mediated by the TIR1/AFB-Aux/IAA-ARF pathway (Leyser 2018). However, increasing evidence indicates that auxin also operates through rapid, non-transcriptional mechanisms that depend on its cytosolic signaling via the AFB1 receptor (Fendrych et al. 2016; Dubey et al. 2023). Beyond intracellular perception, extracellular auxin is perceived at the cell surface in the apoplast by TMK1, a receptor-like kinase, which forms co-receptor complexes with the auxin-binding proteins ABL1 and ABL2. This ABL–TMK1 complex activates downstream ROP GTPases, leading to cellular changes (Xu et al. 2014; Yu et al. 2023). Together, these findings support a dual framework in which rapid auxin responses are mediated both by intracellular AFB1 signaling and by an apoplastic ABL–TMK receptor complex at the plasma membrane.

**Fig. 2:**
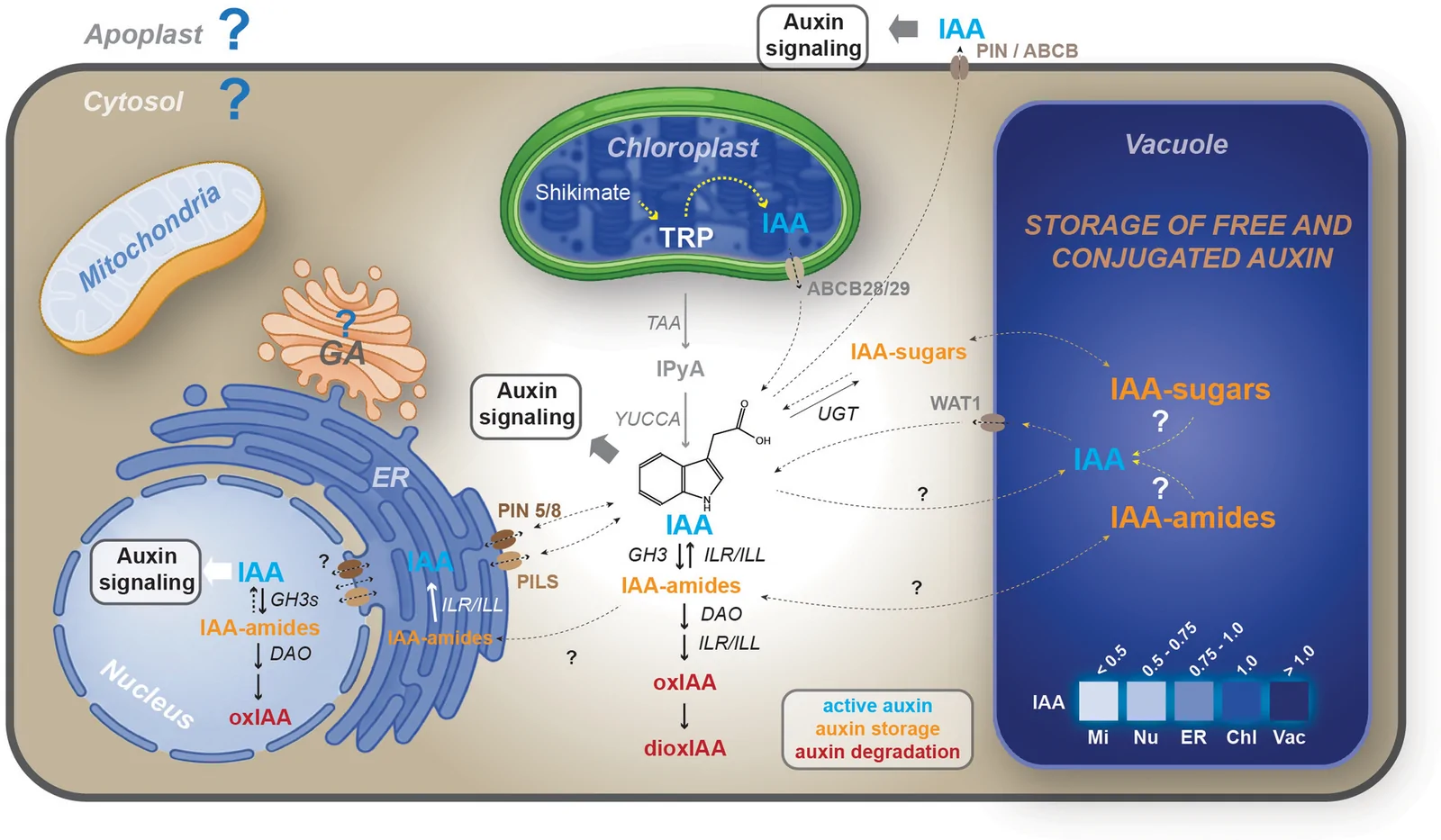
Auxin subcellular distribution and pathways. The relative concentration of IAA is shown by shades of blue in relation to chloroplasts (adapted from Skalický et al. 2023). IAA was determined in the nucleus, endoplasmic reticulum, chloroplasts, mitochondria and vacuoles. IAA biosynthesis begins in the chloroplast with the conversion of shikimate into tryptophan (TRP), followed in Arabidopsis mainly by its transformation into indole-3-pyruvic acid (IPyA) and subsequently into IAA in the cytosol. The resulting free IAA is subjected to tight spatiotemporal regulation via transport, conjugation, storage, and catabolism. Intracellular auxin gradients are shaped by specific transporters such as PIN5/8 and PIN-LIKES (PILS) proteins, which modulate IAA distribution across intracellular membranes, including the endoplasmic reticulum (ER). To regulate active IAA levels, the hormone is conjugated to amino acids (IAA-amides) via GH3 enzymes, or to sugars (IAA-sugars) through UDP-glucosyltransferases (UGTs). These conjugated forms serve as reversible inactivation and storage pools and can be hydrolyzed by specific hydrolases (e.g., ILR1/ILL) to release free IAA on demand. The vacuole plays a critical role in sequestering both free and conjugated IAA, though the precise transporters and trafficking routes involved remain largely unresolved (indicated by question marks). The WAT1 transporter has been implicated in IAA mobilization between the cytosol and the vacuole. Oxidative inactivation, primarily catalyzed by DAO enzymes, converts IAA into irreversible catabolites such as oxIAA and dioxIAA, thereby terminating its hormonal activity. Together, these interconnected pathways enable precise intracellular control of auxin homeostasis, underpinning a wide array of developmental and environmental responses. The figure also highlights current knowledge gaps in auxin distribution and subcellular transport pathways (indicated by question marks), offering clear directions for future research

Historically, progress in studying intracellular phytohormone dynamics has been constrained by the limitations of analytical tools. Moreover, challenges such as sufficient purity of isolated compartments, preventing metabolic changes during isolation, and ensuring compartmental integrity further complicate subcellular hormone profiling. Despite these difficulties, significant advancements in both isolation protocols and analytical performance open new opportunities to investigate intracellular auxin homeostasis. One such breakthrough is the development of Fluorescence-Activated Multi-Organelle Sorting (FAmOS), a subcellular fractionation method based on flow cytometry principles (Skalický et al. 2023) which enables the simultaneous sorting of four distinctly labeled organelles—mitochondria, ER, nuclei, and chloroplasts—while maintaining the metabolic stability of hormones. Furthermore, the authors extended the portfolio of four organelles to five by isolating vacuoles using ultracentrifugation principles. Data obtained using FAmOS combined with LC–MS analysis revealed distinct subcellular distributions of auxin, emphasizing the formation of phytohormone gradients (Fig. 2). Beyond their spatial organization, these gradients may also reflect functional links between hormone homeostasis and cellular energy metabolism. Tivendale and Millar (2022) proposed that auxin biosynthesis, transport, and signaling are coordinated with mitochondrial and chloroplastic functions, thereby linking hormone signaling with respiratory and photosynthetic activity. Within this framework, organelle-specific auxin pools may therefore reflect not only regulatory mechanisms but also local metabolic and energetic demands of individual cellular compartments.

Understanding auxin distribution at the subcellular level goes hand in hand with exploring the mechanisms of auxin transport between cell compartments. To date, auxin carriers have been identified at multiple subcellular locations, including the plasma membrane, ER, vacuole, and chloroplasts. These transport systems collectively shape intracellular auxin distribution and contribute to the establishment of local auxin gradients. The ER, in particular, plays a significant role in auxin subcellular homeostasis, housing key proteins involved in auxin biosynthesis (YUC4.2), conjugate hydrolysis (IAR/ILL2/ILR), and transport.

At the plasma membrane, plant ABCB proteins, a subgroup of ATP-binding cassette transporters, function as ATP-dependent auxin efflux carriers. Members such as ABCB1, ABCB4, and ABCB19 mediate auxin export from the cytosol to the apoplast and contribute to auxin distribution within tissues, as knockout mutants display reduced long-distance auxin transport, altered root growth, and gravitropic defects (Mellor et al. 2022). Although ABCBs can mediate auxin efflux independently, their interaction with PIN proteins enhances transport efficiency and directionality. This synergistic relationship explains why combined loss-of-function mutants often exhibit more severe defects in auxin distribution than single mutants (Cho and Cho 2013; Mellor et al. 2022). Unlike PIN proteins, ABCBs are generally evenly distributed at the plasma membrane and exhibit limited endosomal recycling, suggesting that they provide a basal auxin efflux capacity that complements dynamic PIN-mediated transport patterns. Auxin gradients emerging from the interplay between steady ABCB-mediated efflux and dynamic, polarized PIN transport are central to plant patterning. Stable ABCB activity maintains baseline auxin distribution, whereas PIN polarization and endosomal recycling generate directional auxin fluxes required for tropic responses and organogenesis (Cho et al. 2012; Cho and Cho 2013).

In addition to ABCB transporters providing basal auxin efflux, members of the PIN-FORMED (PIN) family have been key regulators of directional auxin transport and subcellular auxin dynamics. Directional auxin transport primarily depends on the polar localization of PIN proteins, which is established and maintained through continuous clathrin-mediated endocytosis and ARF-GEF–dependent recycling, particularly via GNOM-mediated trafficking (Steinmann et al. 1999; Geldner et al. 2003). This dynamic recycling allows rapid reorientation of auxin flow during developmental and environmental responses. Importantly, auxin itself inhibits PIN endocytosis, promoting plasma membrane retention and reinforcing its own directional transport (Paciorek et al. 2005).

In Arabidopsis, 8 members of the PIN family, systematically named PIN1-PIN8, have been described. Canonical PINs (PIN1, PIN2, PIN3, PIN4, PIN7) characterized by long hydrophilic loop localize to the plasma membrane, while non-canonical members (PIN5, PIN8) localize to the ER. PIN6 is found in both locations. For PIN5, PIN6, and PIN8, a short central hydrophilic loop is characteristic (Ding et al. 2012; Mravec et al. 2009; Simon et al. 2016). PIN5 and PIN8 proteins exhibit antagonistic roles in regulating intracellular auxin homeostasis and development. PIN5 enhances 1-naphthaleneacetic acid (NAA) accumulation in BY-2 tobacco cells, promotes root hair growth, and reduces IAA export from the cytoplasm out of the cell (Mravec et al. 2009; Ganguly et al. 2010). In contrast, PIN8 inhibits root hair growth and increases auxin export from the ER into the cytoplasm and potentially further out of the cell (Ding et al. 2012). These findings suggest that ‘short’ PINs may regulate auxin subcellular homeostasis by transporting auxin between the ER lumen and cytosol, or potentially from the ER lumen to the nucleus.

Middleton et al (2018) showed that ER-to-nucleus auxin transport is a critical mechanism regulating nuclear auxin levels. The authors propose that auxin-mediated responses are controlled by the maintenance of a homeostatic auxin pool within the ER, along with regulated rapid auxin fluxes between the ER and nucleus. The ER-nucleus gradient observed by Skalický et al (2023) supports the hypothesis, suggesting that the ER not only stores auxin but also modulates its delivery for nuclear signaling. This is further reinforced by the activity of ILR-like hydrolases in the ER, which release free IAA from conjugates, potentially supplying the nucleus with active auxin (Sanchez Carranza et al. 2016).

Another group of auxin transporters identified in *Arabidopsis thaliana* and localized to the ER are PIN-LIKES (PILS) proteins (Barbez et al. 2012; Feraru et al. 2012). PILS were named based on their similarity to PIN proteins, however, subsequent comparative study revealed that they share only 10–12% sequence identity and are evolutionarily distinct (Feraru et al. 2012). They regulate nuclear auxin abundance and signaling by reducing auxin diffusion into the nucleus. Environmental factors like light and temperature influence PILS protein levels, adjusting auxin-dependent organ growth accordingly. Posttranslational regulation of PILS proteins can override their transcriptional control, highlighting the importance of PILS turnover in development. However, the cellular mechanisms linking external cues to PILS protein abundance remain largely unknown (Béziat et al. 2017; Feraru et al. 2019).

ER auxin carriers establish a clear link between auxin transport and its metabolic inactivation. Overexpression of PIN5 results in a marked increase in the levels of conjugates IAA-Asp and IAA-Glu, accompanied by a reduction in free IAA concentrations (Mravec et al. 2009). In contrast, overexpression of PIN8 leads to the opposite trend—elevated levels of free IAA and a decrease in conjugated forms (Ding et al. 2012). Consistently, genetic analyses revealed that altered PILS activity shifts the balance between free IAA and its amide conjugates, indicating a direct contribution of ER-localized transport processes to auxin metabolic homeostasis (Barbez et al. 2012). Other studies further demonstrate that the hydrolysis of auxin amide conjugates occurs within the ER and possibly the adjacent cytosol, mediated by ILR-like enzymes (Sanchez Carranza et al. 2016). These findings raise several important questions: What role do transporters play in the regulation of auxin conjugation and degradation? How is the antagonistic interplay between GH3 enzymes and ILR/ILL hydrolases coordinated?

Včelařová et al. (2021) analyzed auxin profiles in ER-enriched fractions isolated from Arabidopsis seedlings. The ER-enriched fraction exhibited significantly higher levels of free IAA, while inactive metabolites such as IAA-Asp, IAA-Glu, IAA-glc, and oxIAA were less abundant. These findings suggest that while the ER may act as a reservoir for free IAA, conjugate formation and accumulation primarily occur in other compartments. The interaction between GH3 and ILR/ILL enzymes may be spatially regulated, with their respective activities occurring in neighboring cellular compartments. This is supported by localizing GH3 enzymes in the cytosol and the nucleus (Di Mambro et al. 2019; Široká et al. 2025). If auxin is actively transported from the ER to the nucleus, the availability of free IAA in the ER could thus influence the transcriptional output of auxin-responsive genes. Localizing ILR/ILL activity to the ER would therefore serve to supply nuclear free IAA, enhancing transcriptional regulation. Together, these insights highlight the ER as a central hub for fine-tuning subcellular auxin homeostasis, ensuring optimal plant growth and development through coordinated transport, conjugation, and hydrolysis processes.

Despite the focus on the ER, nuclear auxin metabolism is also emerging as crucial. A recent study detected IAA biosynthetic precursors (IPyA, IAM, TRA), conjugates (IAA-Asp, IAA-Glu), and oxidative metabolites (oxIAA) within nuclei of *A. thaliana* and tobacco (Skalický et al. 2021). Müller et al. (2021) demonstrated the activity of the DAO enzyme in nucleus, which together with confirmed nuclear localization of GH3s support the notion that auxin inactivation machinery operate in the central organelle, fine-tuning auxin levels directly at its site of action. Oxidized auxin catabolites appear to be exported from the nucleus via unknown transporters.

In addition to ER, another auxin active transporter has been identified in vacuoles named WALLS ARE THIN 1 (WAT1). Located on the membrane of the vacuole, WAT1 likely mediates the efflux of IAA from the vacuole to the cytoplasm. The name WALLS ARE THIN 1 reflects a curious phenotype: plants with a mutation in this gene tend to have weakened cell walls. This suggests WAT1 has a broader role beyond auxin transport, potentially influencing the metabolism of cell wall components, like lignin, which is important for structural support. WAT1 is essential for maintaining auxin homeostasis within the cell. In *wat1* mutants, altered subcellular distribution of auxin results in deregulation of auxin-responsive genes (Ranocha et al. 2013). Furthermore, IAA conjugates with glucose (Ranocha et al. 2013) or amino acids (Skalický et al. 2023) have been detected in vacuoles. Although these conjugates have also been reported in other organelles, their high abundance in vacuoles indicates that this compartment likely serves as an important storage site for excess auxin. These findings further highlight the question of where GH3-mediated IAA conjugation takes a place, given that WAT1 does not transport IAA conjugates (Ranocha et al. 2013). The presence of a transporter for IAA conjugates could support the cytoplasmic localization of GH3 enzymes, as proposed by Di Mambro et al. (2019) and Široká et al. (2025). However, the possibility that some members of the GH3 family may operate in vacuole cannot be excluded. Further research is needed to elucidate auxin metabolism in vacuoles, particularly the mechanism by which auxin enters this organelle. Notably, the highest concentration of free IAA has been detected in vacuoles (Skalický et al. 2023). Since the vacuole is not the primary site of IAA action, its accumulation may serve as a reservoir of bioactive auxin, ensuring a rapid cellular responses in situations where the hydrolysis IAA conjugates would be too slow.

While most studies have focused on the endomembrane system and nucleus, increasing attention has recently been paid to the role of plastids in auxin homeostasis. Recently, auxin efflux transporters were identified in chloroplasts, ABCB28 in inner membrane and ABCB29 in outer membrane (Tamizhselvan et al. 2023). Using stable isotope-labeled indole feeding, authors also proposed chloroplastic biosynthesis of IAA. The proposed Trp-dependent biosynthesis via IPyA resembles biosynthetic routes that have been identified in cyanobacteria (Sergeeva et al. 2002). These findings are further supported by the observations of Skalický et al. (2023), who detected a relatively high concentration of IAA in chloroplasts among the studied organelles (Fig. 2). Although it has been proposed that IAA biosynthesis occurs mainly in the cytoplasm, the presence of a IAA biosynthesis in the chloroplast reflects the endosymbiotic origin of plastids and highlights their potential role in auxin metabolism. In this context, chloroplast-localized auxin metabolism may represent an important link between photosynthetic carbon assimilation, tryptophan biosynthesis, cellular energy status, and auxin-dependent growth regulation. This spatial coupling may enable plants to coordinate developmental signaling with primary metabolism in response to changing metabolic and environmental conditions.

At present, little is known about the role of auxin in mitochondria. Only low levels of IAA, oxIAA, and oxIAA-glc have been detected in this organelle (Skalický et al. 2023), which is insufficient to draw any conclusions regarding their physiological relevance. Moreover, no mitochondria-specific auxin transporters have been identified so far, further limiting our understanding of auxin dynamics in this compartment. In this context, other cellular compartments, such as the Golgi apparatus and cytosol, also remain largely unexplored and represent important targets for future research.

## Conclusion and perspectives

Recent advancements in instrumental techniques continue to expand the frontiers of our understanding of phytohormone dynamics at the (sub)cellular level. The establishment of the first subcellular map of phytohormone distribution (Skalický et al. 2023) represents a significant milestone, offering a valuable framework for unraveling the intricate regulatory networks governing plant hormone function at the subcellular scale. Despite this progress, several fundamental questions remain unresolved—particularly concerning the precise localization and behavior of auxin within distinct cellular compartments. It remains unclear whether the observed auxin pools originate from localized biosynthesis or are shaped through specific transport processes. To construct a more comprehensive and spatially resolved map of auxin distribution, it is imperative to develop more efficient and selective methods for the isolation of intracellular compartments, with particular emphasis on the cytosole. Addressing this challenge will necessitate a multifaceted approach that integrates state-of-the-art bioanalytical platforms with cellular, molecular, biochemical, and proteomic methodologies. Moreover, the recent identification of an auxin receptor localized at the outer surface of the plasma membrane (Yu et al. 2023) further underscores the necessity of distinguishing between intracellular (symplastic) and extracellular (apoplastic) auxin pools. This finding highlights the added complexity of auxin signaling and transport, as well as their potential integration with cellular metabolic and bioenergetic processes, and calls for the development of analytical strategies capable of resolving hormone profiles across the cellular boundary. An important open question is whether intercellular auxin transport and the formation of extracellular auxin pools are also functionally linked to metabolic processes controlling auxin availability and turnover.

Also, auxin biosynthesis is probably not fully understood, which underscores the need for integrative omics approaches to accurately reconstruct the enzymatic pathways leading to IAA. Fenech et al. (2025) concludes that the linear IAOx → IAN → IAM → IAA pathway is not functional in Arabidopsis. While IAOx, IAN, and IAM can each be converted to IAA, they do so via independent pathways. The *sur2* mutant, which accumulates IAOx, overproduces IAA through an unknown mechanism that does not involve the known biosynthetic gene families. Thus, the IPyA pathway remains the dominant and physiologically relevant route for IAA biosynthesis in plants. In addition, the tryptophan-independent pathway remains controversial, as no conclusive enzymes or genes have been identified to support its existence.

Looking ahead, the future of phytohormone research is poised for substantial progress. Continued improvements in analytical resolution and the implementation of innovative methodological approaches are expected to yield new insights into auxin metabolism and its spatial dynamics within plant tissues, cells, and organelles. Ultimately, a deeper understanding of these processes will enhance our knowledge of plant developmental biology and offer novel avenues for optimizing crop performance and stress resilience through targeted modulation of hormone signaling pathways.

## Data Availability

Not applicable.

## References

[CR1] Ashen JB, Cohen JD, Goff LJ (1999) GC-SIM-MS detection and quantification of free indole-3-acetic acid in bacterial galls on the marine alga *Prionitis lanceolata* (Rhodophyta). J Phycol 35:493–500. 10.1046/j.1529-8817.1999.3530493.x

[CR2] Ashton NW, Schulze A, Hall P, Bandurski RS (1985) Estimation of indole-3-acetic acid in gametophytes of the moss *Physcomitrella patens*. Planta 164:142–144. 10.1007/BF00391040 11540856

[CR3] Bandurski RS, Ueda M, Nicholls PB (1969) Esters of indole-3-acetic acid and *myo*-inositol. Ann N Y Acad Sci 165:655–667. 10.1111/j.1749-6632.1970.tb56432.x

[CR4] Barbez E, Kubeš M, Rolčík J, Béziat C, Pěnčík A, Wang B, Rosquete MR, Zhu J, Dobrev PI, Lee Y, Zažímalová E, Petrášek J, Geisler M, Friml J, Kleine-Vehn J (2012) A novel putative auxin carrier family regulates intracellular auxin homeostasis in plants. Nature 485:119–122. 10.1038/nature11001 22504182

[CR5] Bartel B, LeClere S, Magidin M, Zolman BK (2001) Inputs to the active indole-3-acetic acid pool: de novo synthesis, conjugate hydrolysis, and indole-3-butyric acid β-oxidation. J Plant Growth Regul 20:198–216. 10.1007/s003440010025

[CR6] Basse CW, Lottspeich F, Steglich W, Kahmann R (1996) Two potential indole-3-acetaldehyde dehydrogenases in the phytopathogenic fungus *Ustilago maydis*. Eur J Biochem 242:648–656. 10.1111/j.1432-1033.1996.0648r.x 9022693

[CR7] Béziat C, Barbez E, Feraru MI, Lucyshyn D, Kleine-Vehn J (2017) Light triggers PILS-dependent reduction in nuclear auxin signalling for growth transition. Nat Plants 3:17105. 10.1038/nplants.2017.105 28714973 PMC5524181

[CR8] Bierfreund NM, Tintelnot S, Reski R, Decker EL (2004) Loss of GH3 function does not affect phytochrome-mediated development in a moss, *Physcomitrella patens*. J Plant Physiol 161:823–835. 10.1016/j.jplph.2003.12.010 15310072

[CR9] Brunoni F, Collani S, Casanova-Sáez R, Šimura J, Karady M, Schmid M, Ljung K, Bellini C (2020) Conifers exhibit a characteristic inactivation of auxin to maintain tissue homeostasis. New Phytol 226:1753–1765. 10.1111/nph.16463 32004385

[CR10] Brunoni F, Pěnčík A, Žukauskaitė A, Ament A, Kopečná M, Collani S, Kopečný D, Novák O (2023) Amino acid conjugation of oxIAA is a secondary metabolic regulation involved in auxin homeostasis. New Phytol 238:2264–2270. 10.1111/nph.18887 36941219

[CR11] Campanella JJ, Olajide AF, Magnus V, Ludwig-Müller J (2004) A novel auxin conjugate hydrolase from wheat with substrate specificity for longer side-chain auxin amide conjugates. Plant Physiol 135:2230–2240. 10.1104/pp.104.043398 15299127 PMC520793

[CR12] Campanella JJ, Kurdach S, Bochis J, Smalley JV (2018) Evidence for exaptation of the *Marchantia polymorpha* M20D peptidase MpILR1 into the tracheophyte auxin regulatory pathway. Plant Physiol 177:1595–1604. 10.1104/pp.18.00543 29959171 PMC6084679

[CR13] Carrillo-Carrasco VP, Hernandez-Garcia J, Mutte SK, Weijers D (2023) The birth of a giant: evolutionary insights into the origin of auxin responses in plants. EMBO J 42:EMBJ2022113018. 10.15252/embj.2022113018 PMC10015382 36786017

[CR14] Casanova-Sáez R, Mateo-Bonmatí E, Šimura J, Pěnčík A, Novák O, Staswick P, Ljung K (2022) Inactivation of the entire *Arabidopsis* group II GH3s confers tolerance to salinity and water deficit. New Phytol 235:263–275. 10.1111/nph.18114 35322877 PMC9322293

[CR15] Cavallari N, Artner C, Benková E (2021) Auxin-regulated lateral root organogenesis. Cold Spring Harb Perspect Biol 13:a039941. 10.1101/cshperspect.a039941 33558367 PMC8247565

[CR16] Chen Q, Westfall CS, Hicks LM, Wang S, Jez JM (2010) Kinetic basis for the conjugation of auxin by a GH3 family indole-acetic acid–amido synthetase. J Biol Chem 285:29780–29786. 10.1074/jbc.M110.146431 20639576 PMC2943290

[CR17] Chen CY, Selvaraj P, Naqvi NI (2023) Functional analysis of auxin derived from a symbiotic mycobiont. Front Plant Sci 14:1216680. 10.3389/fpls.2023.1216680 37745999 PMC10515717

[CR18] Cheng Y, Zhao Y (2007) A role for auxin in flower development. J Integr Plant Biol 49:99–104. 10.1111/j.1744-7909.2006.00412.x

[CR19] Cheng X, Li X, Tong M, Wu J, Chan LL, Cai Z, Zhou J (2023) Indole-3-acetic acid as a cross-talking molecule in algal–bacterial interactions and a potential driving force in algal bloom formation. Front Microbiol 14:1236925. 10.3389/fmicb.2023.1236925 37928680 PMC10623134

[CR20] Cho M, Cho H (2013) The function of ABCB transporters in auxin transport. Plant Signal Behav 8(2):e22990. 10.4161/psb.22990 23221777 PMC3656995

[CR21] Cho M, Lee ZW, Cho HT (2012) ATP-binding cassette B4, an auxin-efflux transporter, stably associates with the plasma membrane and shows distinctive intracellular trafficking from that of PIN-FORMED proteins. Plant Physiol 159(2):642–654. 10.1104/pp.112.196139 22492845 PMC3375931

[CR22] Ciarkowska A, Wojtaczka P, Kęsy J, Ostrowski M (2022) Auxin homeostasis in maize (*Zea mays*) is regulated via 1-O-indole-3-acetyl-myo-inositol synthesis at early stages of seedling development and under abiotic stress. Planta 257:23. 10.1007/s00425-022-04058-z 36539632 PMC9768015

[CR23] Ciesielski T (1872) Untersuchungen über die Abwärtskrümmung der Wurzel. Beitr Biol Pflanz 1:1–31

[CR24] Darwin C, Darwin F (1880) The power of movement in plants. Murray, London

[CR25] Di Mambro R, Svolacchia N, Dello Ioio R, Pierdonati E, Salvi E, Pedrazzini E, Vitale A, Perilli S, Sozzani R, Benfey PN, Busch W, Costantino P, Sabatini S (2019) The lateral root cap acts as an auxin sink that controls meristem size. Curr Biol 29:1199–1205. 10.1016/j.cub.2019.02.022 30880016

[CR26] Ding Z, Wang B, Moreno I, Dupláková N, Simon S, Carraro N, Reemmer J, Pěnčík A, Chen X, Tejos R, Skůpa P, Pollmann S, Mravec J, Petrášek J, Zažímalová E, Honys D, Rolčík J, Murphy A, Orellana A, Geisler M, Friml J (2012) ER-localized auxin transporter PIN8 regulates auxin homeostasis and male gametophyte development in *Arabidopsis*. Nat Commun 3:941. 10.1038/ncomms1941 22760640

[CR27] Dobrev P, Filepova R, Lacek J, Vondráková Z, Muller K, Marsik P, Drasarova L, Talacko P, Hošek P, Petrasek J (2023) Study of auxin metabolism using stable isotope labeling and LCMS evidence for in planta auxin decarboxylation pathway. bioRxiv. 10.1101/2023.06.02.54338437292708

[CR28] Drábková LZ, Dobrev PI, Motyka V (2015) Phytohormone profiling across the bryophytes. PLoS ONE 10(5):e0125411. 10.1371/journal.pone.0125411 25974061 PMC4431756

[CR29] Dubey SM, Han S, Stutzman N, Prigge MJ, Medvecká E, Platre MP, Busch W, Fendrych M, Estelle M (2023) The AFB1 auxin receptor controls the cytoplasmic auxin response pathway in *Arabidopsis thaliana*. Mol Plant 16(7):1120–1130. 10.1016/j.molp.2023.06.008 37391902 PMC10720607

[CR30] Eklund DM, Ishizaki K, Flores-Sandoval E, Kikuchi S, Takebayashi Y, Tsukamoto S, Hirakawa Y, Nonomura M, Kato H, Kouno M, Bhalerao RP, Lagercrantz U, Kasahara H, Kohchi J, Bowman JL (2015) Auxin produced by the indole-3-pyruvic acid pathway regulates development and gemmae dormancy in *Marchantia polymorpha*. Plant Cell 27:1650–1669. 10.1105/tpc.15.00065 26036256 PMC4498201

[CR31] Fendrych M, Leung J, Friml J (2016) TIR1/AFB-Aux/IAA auxin perception mediates rapid cell wall acidification and growth of *Arabidopsis* hypocotyls. Elife 5:19048 10.7554/eLife.19048 PMC5045290 27627746

[CR32] Fenech M, Brumos J, Pěnčík A, Edwards B, Belcapo S, DeLacey J, Patel A, Kater M, Li X, Ljung K, Novak O, Alonso JM, Stepanova AN (2025) The CYP71A, NIT, AMI, and IAMH gene families are dispensable for indole-3-acetaldoxime-mediated auxin biosynthesis in *Arabidopsis*. Plant Cell 37:koaf242. 10.1093/plcell/koaf242 41092117 PMC12586335

[CR33] Feng S, Yue R, Tao S, Yang Y, Zhang L, Xu M, Wang H, Shen C (2015) Genome-wide identification, expression analysis of auxin-responsive GH3 family genes in maize (*Zea mays* L.) under abiotic stresses. J Integr Plant Biol 57:783–795. 10.1111/jipb.12327 25557253

[CR34] Feraru E, Vosolsobě S, Feraru MI, Petrášek J, Kleine-Vehn J (2012) Evolution and structural diversification of PILS putative auxin carriers in plants. Front Plant Sci 3:227. 10.3389/fpls.2012.00227 23091477 PMC3470039

[CR35] Feraru E, Feraru MI, Barbez E, Waidmann S, Sun L, Gaidora A, Kleine-Vehn J (2019) PILS6 is a temperature-sensitive regulator of nuclear auxin input and organ growth in *Arabidopsis thaliana*. Proc Natl Acad Sci USA 116:3893–3898. 10.1073/pnas.1814015116 30755525 PMC6397578

[CR36] Frick EM, Strader LC (2018) Roles for IBA-derived auxin in plant development. J Exp Bot 69:169–177. 10.1093/jxb/erx298 28992091 PMC5853464

[CR37] Fu X, Shi Z, Jiang Y, Jiang L, Qi M, Xu T, Li T (2019) A family of auxin conjugate hydrolases from *Solanum lycopersicum* and analysis of their roles in flower pedicel abscission. BMC Plant Biol 19:223. 10.1186/s12870-019-1840-9 31159738 PMC6547480

[CR38] Ganguly A, Lee SH, Cho M, Lee OR, Yoo H, Cho HT (2010) Differential auxin-transporting activities of PIN-FORMED proteins in *Arabidopsis* root hair cells. Plant Physiol 153:1046–1061. 10.1104/pp.110.156505 20439545 PMC2899906

[CR39] Geldner N, Anders N, Wolters H, Keicher J, Kornberger W, Muller P, Delbarre A, Ueda T, Nakano A, Jürgens G (2003) The *Arabidopsis* GNOM ARF-GEF mediates endosomal recycling, auxin transport, and auxin-dependent plant growth. Cell 112(2):219–230. 10.1016/S0092-8674(03)00003-5 12553910

[CR40] Haagen-Smit AJ, Dandliker WB, Wittwer SH, Murneek AE (1946) Isolation of 3-indoleacetic acid from immature corn kernels. Am J Bot 33:118–120. 10.2307/2437327

[CR41] Hagen G, Kleinschmidt A, Guilfoyle T (1984) Auxin-regulated gene expression in intact soybean hypocotyl and excised hypocotyl sections. Planta 162:147–153. 10.1007/bf00410211 24254049

[CR42] Hayashi K (2012) The interaction and integration of auxin signaling components. Plant Cell Physiol 53:965–975. 10.1093/pcp/pcs035 22433459

[CR43] Hayashi K, Arai K, Aoi Y, Tanaka Y, Hira H, Guo R, Hu Y, Ge C, Zhao Y, Kasahara H, Fukui K (2021) The main oxidative inactivation pathway of the plant hormone auxin. Nat Commun 12:6752. 10.1038/s41467-021-27020-1 34811366 PMC8608799

[CR44] Hladík P, Petřík I, Žukauskaitė A, Novák O, Pěnčík A (2023) Metabolic profiles of 2-oxindole-3-acetyl-amino acid conjugates differ in various plant species. Front Plant Sci 14:1217421. 10.3389/fpls.2023.1217421 37534287 PMC10390838

[CR45] Hui S, Zhang M, Hao M, Yuan M (2019) Rice group I GH3 gene family, positive regulators of bacterial pathogens. Plant Signal Behav 14:e1588659. 10.1080/15592324.2019.1588659 30900505 PMC6512922

[CR46] Ishimaru K, Hirotsu N, Madoka Y, Murakami N, Hara N, Onodera H, Kashiwagi T, Ujiie K, Shimizu B, Onishi A, Miyagawa H, Katoh E (2013) Loss of function of the IAA-glucose hydrolase gene TGW6 enhances rice grain weight and increases yield. Nat Genet 45:707–711. 10.1038/ng.2612 23583977

[CR47] Isobe T, Miyagawa H (2022) Facilitation of auxin biosynthesis and metabolism by salt stress in rice plants. Biosci Biotechnol Biochem 86:824–831. 10.1093/bbb/zbac070 35580591

[CR48] Jackson RG, Lim EK, Li Y, Kowalczyk M, Sandberg G, Hoggett J, Ashford DA, Bowles DJ (2001) Identification and biochemical characterization of an *Arabidopsis* indole-3-acetic acid glucosyltransferase. J Biol Chem 276:4350–4356. 10.1074/jbc.m006185200 11042207

[CR49] Jawahir V, Zolman BK (2021) Long chain acyl CoA synthetase 4 catalyzes the first step in peroxisomal indole-3-butyric acid to IAA conversion. Plant Physiol 185:120–136. 10.1093/plphys/kiaa002 33631795 PMC8133310

[CR50] Jin SH, Ma XM, Han P, Wang B, Sun YG, Zhang GZ, Li YJ, Hou BK (2013) UGT74D1 is a novel auxin glycosyltransferase from *Arabidopsis thaliana*. PLoS ONE 8:e61705. 10.1371/journal.pone.0061705 23613909 PMC3628222

[CR51] Kai K, Wakasa K, Miyagawa H (2007) Metabolism of indole-3-acetic acid in rice: identification and characterization of N-beta-D-glucopyranosyl indole-3-acetic acid and its conjugates. Phytochemistry 68:2512–2522. 10.1016/j.phytochem.2007.05.040 17628621

[CR52] Kögl F, Haagen-Smit AJ (1931) Über die Chemie des Wuchsstoffs. Proc Kon Akad Wetensch 34:1411–1416

[CR53] Kögl F, Erxleben H, Haagen-Smit AJ (1934a) Über die Isolierung der Auxine a und b aus pflanzlichen Materialien. Z Physiol Chem 243:209–226

[CR54] Kögl F, Haagen-Smit AJ, Erxleben H (1934b) Über ein neues Auxin (Heteroauxin) aus Harn. XI. Mitteilung über pflanzliche Wachstumsstoffe. Hoppe-Seyler’s Z Physiol Chem 228:90–103

[CR55] Konstantinova N, Korbei B, Luschnig C (2021) Auxin and root gravitropism: addressing basic cellular processes by exploiting a defined growth response. Int J Mol Sci 22:2749. 10.3390/ijms22052749 33803128 PMC7963156

[CR56] Koochak H, Ludwig-Müller J (2021) *Physcomitrium patens* mutants in auxin conjugating GH3 proteins show salt stress tolerance but auxin homeostasis is not involved in regulation of oxidative stress factors. Plants (Basel) 10:1398. 10.3390/plants10071398 34371602 PMC8309278

[CR57] Korasick DA, Enders TA, Strader LC (2013) Auxin biosynthesis and storage forms. J Exp Bot 64:2541–2555. 10.1093/jxb/ert138 23580748 PMC3695655

[CR58] Kowalczyk M, Sandberg G (2001) Quantitative analysis of indole-3-acetic acid metabolites in *Arabidopsis thaliana*. Plant Physiol 127:1845–1853. 10.1104/pp.010525 11743128 PMC133588

[CR59] Kriechbaumer V (2016) ER microsome preparation and subsequent IAA quantification in maize coleoptile and primary root tissue. Bio Protoc 6:e1805. 10.21769/BioProtoc.1805 28180138 PMC5293169

[CR60] LeClere S, Tellez R, Rampey RA, Matsuda SP, Bartel B (2002) Characterization of a family of IAA-amino acid conjugate hydrolases from *Arabidopsis*. J Biol Chem 277:20446–20452. 10.1074/jbc.m111955200 11923288

[CR61] Lewer P, Bandurski RS (1987) Occurrence and metabolism of 7-hydroxy-2-indolinone-3-acetic acid in *Zea mays*. Phytochemistry 26:1247–1250. 10.1016/s0031-9422(00)81790-2 11539052

[CR62] Leyser O (2018) Auxin signaling. Plant Physiol 176:465–479. 10.1104/pp.17.00765 28818861 PMC5761761

[CR63] Ljung K, Ostin A, Lioussanne L, Sandberg G (2001) Developmental regulation of indole-3-acetic acid turnover in Scots pine seedlings. Plant Physiol 125:464–475. 10.1104/pp.125.1.464 11154354 PMC61027

[CR64] Ludwig-Müller J (2007) Indole-3-butyric acid synthesis in ecotypes and mutants of Arabidopsis thaliana under different growth conditions. J Plant Physiol 164:47–59. 10.1016/j.jplph.2005.10.008 16325963

[CR65] Ludwig-Müller J (2011) Auxin conjugates: their role for plant development and in the evolution of land plants. J Exp Bot 62:1757–1773. 10.1093/jxb/erq412 21307383

[CR66] Ludwig-Müller J, Epstein E (1992) Indole-3-acetic acid is converted to indole-3-butyric acid by seedlings of Zea mays L. In: Karssen, CM, van Loon LC, Vreugdenhil D (eds) Progress in Plant Growth Regulation. Current Plant Science and Biotechnology in Agriculture, Vol 13. Springer, Dordrecht. 10.1007/978-94-011-2458-4_20

[CR67] Ludwig-Müller J, Hilgenberg W (1995) Characterization and partial purification of indole-3-butyric acid synthetase from maize (*Zea mays*). Physiol Plant 94:651–660. 10.1111/j.1399-3054.1995.tb00980.x

[CR68] Ludwig-Müller J, Julke S, Bierfreund NM, Decker EL, Reski R (2009) Moss (*Physcomitrella patens*) GH3 proteins act in auxin homeostasis. New Phytol 181:323–338. 10.1111/j.1469-8137.2008.02677.x 19032442

[CR69] Mashiguchi K, Tanaka K, Sakai T, Sugawara S, Kawaide H, Natsume M, Hanada A, Yaeno T, Shirasu K, Yao H, McSteen P, Zhao Y, Hayashi K, Kamiya Y, Kasahara H (2011) The main auxin biosynthesis pathway in Arabidopsis. Proc Natl Acad Sci USA 108:18512–18517. 10.1073/pnas.1108434108 22025724 PMC3215075

[CR70] Mateo-Bonmatí E, Casanova-Sáez R, Šimura J, Ljung K (2021) Broadening the roles of UDP-glycosyltransferases in auxin homeostasis and plant development. New Phytol 232:642–654. 10.1111/nph.17633 34289137

[CR71] Matsuda F, Miyazawa H, Wakasa K, Miyagawa H (2005) Quantification of indole-3-acetic acid and amino acid conjugates in rice by liquid chromatography–electrospray ionization–tandem mass spectrometry. Biosci Biotechnol Biochem 69:778–783. 10.1271/BBB.69.778 15849417

[CR72] Mellor NL, Voß U, Ware A, Janes G, Barrack D, Bishopp A, Bennett AJ, Geisler M, Wells DM, Band LR (2022) Systems approaches reveal that ABCB and PIN proteins mediate co-dependent auxin efflux. Plant Cell 34:2309–2327. 10.1093/plcell/koac086 35302640 PMC9134068

[CR73] Middleton AM, Dal Bosco C, Chlap P, Bensch R, Harz H, Ren F, Bergmann S, Wend S, Weber W, Hayashi KI, Zurbriggen MD, Uhl R, Ronneberger O, Palme K, Fleck C, Dovzhenko A (2018) Data-driven modeling of intracellular auxin fluxes indicates a dominant role of the ER in controlling nuclear auxin uptake. Cell Rep 22:3044–3057. 10.1016/j.celrep.2018.02.074 29539430

[CR74] Mravec J, Skůpa P, Bailly A, Hoyerová K, Křeček P, Bielach A, Petrášek J, Zhang J, Gaykova V, Stierhof YD, Dobrev PI, Schwarzerová K, Rolčík J, Seifertová D, Luschnig C, Benková E, Zažímalová E, Geisler M, Friml J (2009) Subcellular homeostasis of phytohormone auxin is mediated by the ER-localized PIN5 transporter. Nature 459:1136–1140. 10.1038/nature08066 19506555

[CR75] Müller K, Dobrev PI, Pěnčík A, Hošek P, Vondráková Z, Filepová R, Malínská K, Brunoni F, Helusová L, Moravec T, Retzer K, Harant K, Novák O, Hoyerová K, Petrášek J (2021) DIOXYGENASE FOR AUXIN OXIDATION 1 catalyzes the oxidation of IAA amino acid conjugates. Plant Physiol 187:103–115. 10.1093/plphys/kiab242 34618129 PMC8418401

[CR76] Nonhebel HM (2015) Tryptophan-independent indole-3-acetic acid synthesis: critical evaluation of the evidence. Plant Physiol 169:1001–1005. 10.1104/pp.15.01091 26251310 PMC4587473

[CR77] Normanly J, Cohen JD, Fink GR (1993) *Arabidopsis thaliana* auxotrophs reveal a tryptophan-independent biosynthetic pathway for indole-3-acetic acid. Proc Natl Acad Sci U S A 90:10355–10359. 10.1073/pnas.90.21.10355 8234297 PMC47773

[CR78] Novák O, Hényková E, Sairanen I, Kowalczyk M, Pospíšil T, Ljung K (2012) Tissue-specific profiling of the *Arabidopsis thaliana* auxin metabolome. Plant J 72:523–536. 10.1111/j.1365-313x.2012.05085.x 22725617

[CR79] Östin A, Kowalczyk M, Bhalerao RP, Sandberg G (1998) Metabolism of indole-3-acetic acid in Arabidopsis. Plant Physiol 118:285–296. 10.1104/pp.118.1.285 9733548 PMC34867

[CR80] Ostrowski M, Ciarkowska A, Jakubowska A (2016) The auxin conjugate indole-3-acetyl-aspartate affects responses to cadmium and salt stress in *Pisum sativum* L. J Plant Physiol 191:63–72. 10.1016/j.jplph.2015.11.012 26717013

[CR81] Paciorek T, Zažímalová E, Ruthardt N, Petrášek J, Stierhof YD, Kleine-Vehn J, Morris DA, Emans N, Jürgens G, Geldner N, Friml J (2005) Auxin inhibits endocytosis and promotes its own efflux from cells. Nature 435:1251–1256. 10.1038/nature03633 15988527

[CR82] Pěnčík A, Rolčík J, Novák O, Magnus V, Barták P, Buchtík R, Salopek-Sondi B, Strnad M (2009) Isolation of novel indole-3-acetic acid conjugates by immunoaffinity extraction. Talanta 80:651–655. 10.1016/j.talanta.2009.07.043 19836533

[CR83] Pěnčík A, Simonovik B, Petersson SV, Henyková E, Simon S, Greenham K, Zhang Y, Kowalczyk M, Estelle M, Zažímalová E, Novák O, Sandberg G, Ljung K (2013) Regulation of auxin homeostasis and gradients in *Arabidopsis* roots through the formation of the indole-3-acetic acid catabolite 2-oxindole-3-acetic acid. Plant Cell 25:3858–3870. 10.1105/tpc.113.114421 24163311 PMC3877806

[CR84] Pintado A, Hilario DC, Pastor V, Vincent M, Lee SG, Flors V, Ramos C (2023) Allelic variation in the indoleacetic acid-lysine synthase gene of the bacterial pathogen Pseudomonas savastanoi and its role in auxin production. Front Plant Sci 14:123456. 10.3389/fpls.2023.1176705 PMC10280071 37346122

[CR130] Porco S, Pěnčík A, Rashed A, Voss U, Casanova-Sáez R, Bishopp A, Golebiowska A, Bhosale R, Swarup R, Swarup K, Peňáková P (2016) Dioxygenase-encoding AtDAO1 gene controls IAA oxidation and homeostasis in Arabidopsis significance. Proc Natl Acad Sci 113(39):11016–11021. 10.1073/pnas.1604375113 27651491 PMC5047156

[CR85] Poulet A, Kriechbaumer V (2017) Bioinformatics analysis of phylogeny and transcription of TAA/YUC auxin biosynthetic genes. Int J Mol Sci 18:1791. 10.3390/ijms18081791 28820425 PMC5578179

[CR86] Přikryl Z, Vančura V, Wurst M (1985) Auxin formation by rhizosphere bacteria as a factor of root growth. Biol Plant 27:159–163. 10.1007/BF02902155

[CR87] Quittenden LJ, Davies NW, Smith JA, Molesworth PP, Tivendale ND, Ross JJ (2009) Auxin biosynthesis in pea: characterization of the tryptamine pathway. Plant Physiol 151:1130–1138. 10.1104/pp.109.141507 19710233 PMC2773097

[CR88] Ranocha P, Dima O, Nagy R, Felten J, Corratgé-Faillie C, Novák O, Morreel K, Lacombe B, Martinez Y, Pfrunder S, Jin X, Renou JP, Thibaud JB, Ljung K, Fischer U, Martinoia E, Boerjan W, Goffner D (2013) *Arabidopsis* WAT1 is a vacuolar auxin transport facilitator required for auxin homoeostasis. Nat Commun 4:2625. 10.1038/ncomms3625 24129639 PMC3826630

[CR89] Reinecke DM, Bandurski RS (1981) Metabolic conversion of 14C-indole-3-acetic acid to 14C-oxindole-3-acetic acid. Biochem Biophys Res Commun 103:429–433. 10.1016/0006-291X(81)90470-8 7036993

[CR90] Roychoudhry S, Kepinski S (2022) Auxin in root development. Cold Spring Harb Perspect Biol 14:a039933. 10.1101/cshperspect.a039933 34312248 PMC9121899

[CR91] Sanchez Carranza AP, Singh A, Steinberger K, Panigrahi K, Palme K, Dovzhenko A, Dal Bosco C (2016) Hydrolases of the ILR1-like family of *Arabidopsis thaliana* modulate auxin response by regulating auxin homeostasis in the endoplasmic reticulum. Sci Rep 6:24212. 10.1038/srep24212 27063913 PMC4827090

[CR92] Savić B, Tomić S, Magnus V, Gruden K, Barle K, Grenković R, Ludwig-Müller J, Salopek-Sondi B (2009) Auxin amidohydrolases from *Brassica rapa* cleave the alanine conjugate of indolepropionic acid as a preferable substrate: a biochemical and modeling approach. Plant Cell Physiol 50:1587–1599. 10.1093/pcp/pcp101 19602499

[CR93] Sergeeva E, Liaimer A, Bergman B (2002) Evidence for production of the phytohormone indole-3-acetic acid by cyanobacteria. Planta 215:229–238. 10.1007/s00425-002-0749-x 12029472

[CR94] Sherp AM, Westfall CS, Alvarez S, Jez JM (2018) *Arabidopsis thaliana* GH3.15 acyl acid amido synthetase has a highly specific substrate preference for the auxin precursor indole-3-butyric acid. J Biol Chem 293:4277–4288. 10.1074/jbc.ra118.002006 29462792 PMC5868247

[CR95] Simon S, Skůpa P, Viaene T, Zwiewka M, Tejos R, Klíma P, Čarná M, Rolčík J, De Rycke R, Moreno I, Dobrev PI, Orellana A, Zažímalová E, Friml J (2016) PIN6 auxin transporter at endoplasmic reticulum and plasma membrane mediates auxin homeostasis and organogenesis in Arabidopsis. New Phytol 211:65–74. 10.1111/nph.14019 27240710

[CR96] Široká J, Ament A, Mik V, Pospíšil T, Kralová M, Zhang C, Pernisová M, Karady M, Nožková V, Nishizato Y, Kaji T, Saito R, Htitich M, Floková K, Wasternack C, Strnad M, Ueda M, Novák O, Brunoni F (2025) Amide conjugates of the jasmonate precursor cis-(+)-12-oxo-phytodienoic acid regulate its homeostasis during plant stress responses. Plant Physiol 197:1–20. 10.1093/plphys/kiae636 PMC11663710 39607728

[CR97] Skalický V, Vojtková T, Pěnčík A, Vrána J, Juzoń K, Koláčková V, Sedlářová M, Kubeš MF, Novák O (2021) Auxin metabolite profiling in isolated and intact plant nuclei. Int J Mol Sci 22:12369. 10.3390/ijms222212369 34830250 PMC8620152

[CR98] Skalický V, Antoniadi I, Pěnčík A, Chamrád I, Lenobel R, Kubeš MF, Zatloukal M, Žukauskaitė A, Strnad M, Ljung K, Novák O (2023) Fluorescence-activated multi-organelle mapping of subcellular plant hormone distribution. Plant J 116:1825–1841. 10.1111/tpj.16456 37682018

[CR99] Spaepen S, Vanderleyden J, Remans R (2007) Indole-3-acetic acid in microbial and microorganism-plant signaling. FEMS Microbiol Rev 31:425–448. 10.1111/j.1574-6976.2007.00072.x 17509086

[CR100] Staswick PE (2009) The tryptophan conjugates of jasmonic and indole-3-acetic acids are endogenous auxin inhibitors. Plant Physiol 150:1310–1321. 10.1104/pp.109.138529 19458116 PMC2705031

[CR129] Staswick PE, Serban B, Rowe M, Tiryaki I, Maldonado MT, Maldonado MC, Suza W (2005) Characterization of an arabidopsis enzyme family that conjugates amino acids to indole-3-acetic acid. Abstr Plant Cell 17(2):616–627. 10.1105/tpc.104.026690 PMC548830 15659623

[CR101] Steinmann T, Geldner N, Grebe M, Mangold S, Jackson CL, Paris S, Gälweiler L, Palme K, Jürgens G (1999) Coordinated polar localization of auxin efflux carrier PIN1 by GNOM ARF GEF. Science 286(5438):316–318. 10.1126/science.286.5438.316 10514379

[CR102] Stepanova AN, Robertson-Hoyt J, Yun J, Benavente LM, Xie DY, Doležal K, Schlereth A, Jürgens G, Alonso JM (2008) TAA1-mediated auxin biosynthesis is essential for hormone crosstalk and plant development. Cell 133:177–191. 10.1016/j.cell.2008.01.047 18394997

[CR103] Stepanova AN, Yun J, Robles LM, Novak O, He W, Guo H, Ljung K, Alonso JM (2011) The *Arabidopsis YUCCA1* flavin monooxygenase functions in the indole-3-pyruvic acid branch of auxin biosynthesis. Plant Cell 23:3961–3973. 10.1105/tpc.111.088047 22108406 PMC3246335

[CR104] Stirk WA, Ördög V, Novák O, Rolčík J, Strnad M, Bálint P, van Staden J (2013) Auxin and cytokinin relationships in 24 microalgal strains. J Phycol 49:459–467. 10.1111/jpy.12061 27007035

[CR105] Strader LC, Hendrickson Culler A, Cohen JD, Bartel B (2010) Conversion of endogenous indole-3-butyric acid to indole-3-acetic acid drives cell expansion in *Arabidopsis* seedlings. Plant Physiol 153:1577–1586. 10.1104/pp.110.157461 20562230 PMC2923913

[CR106] Strader LC, Wheeler DL, Christensen SE, Berens JC, Cohen JD, Rampey RA, Bartel B (2011) Multiple facets of *Arabidopsis* seedling development require indole-3-butyric acid-derived auxin. Plant Cell 23:984–999. 10.1105/tpc.111.083071 21406624 PMC3082277

[CR107] Szerszen JB, Szczyglowski K, Bandurski RS (1994) *Iaglu*, a gene from *Zea mays* involved in conjugation of growth hormone indole-3-acetic acid. Science 265:1699–1701. 10.1126/science.8085154 8085154

[CR108] Tam YY, Epstein E, Normanly J (2000) Characterization of auxin conjugates in *Arabidopsis*: low steady-state levels of indole-3-acetyl-aspartate, indole-3-acetyl-glutamate, and indole-3-acetyl-glucose. Plant Physiol 123:589–596. 10.1104/pp.123.2.589 10859188 PMC59026

[CR109] Tamizhselvan P, Madhavan S, Constan-Aguilar C, Elrefaay ER, Liu J, Pěnčík A, Novák O, Cairó A, Hrtyan M, Geisler M, Tognetti VB (2023) Chloroplast auxin efflux mediated by *ABCB28* and *ABCB29* fine-tunes salt and drought stress responses in *Arabidopsis*. Plants 13:7. 10.3390/plants13010007 38202315 PMC10780339

[CR110] Tanaka E, Koga H, Mori M, Masashi M (2011) Auxin production by the rice blast fungus and its localization in host tissue. J Phytopathol 159:522–530. 10.1111/j.1439-0434.2011.01799.x

[CR111] Tanaka K, Hayashi K, Natsume M, Kamiya Y, Sakakibara H, Kawaide H, Kasahara H (2014) *UGT74D1* catalyzes the glucosylation of 2-oxindole-3-acetic acid in the auxin metabolic pathway in *Arabidopsis*. Plant Cell Physiol 55:218–228. 10.1093/pcp/pct173 24285754 PMC3894777

[CR112] Tao Y, Ferrer JL, Ljung K, Pojer F, Hong F, Long JA, Li L, Moreno JE, Bowman ME, Ivans LJ, Cheng Y, Lim J, Zhao Y, Ballaré CL, Sandberg G, Noel JP, Chory J (2008) Rapid synthesis of auxin via a new tryptophan-dependent pathway is required for shade avoidance in plants. Cell 133:164–176. 10.1016/j.cell.2008.01.049 18394996 PMC2442466

[CR113] Thimann KV (1935) On the plant growth hormone produced by *Rhizopus suinus*. J Biol Chem 109:279–291. 10.1016/S0021-9258(18)54321-0

[CR114] Tivendale ND, Millar AH (2022) How is auxin linked with cellular energy pathways to promote growth? New Phytol 233:2397–2404. 10.1111/nph.17946 34984715

[CR115] Tivendale ND, Davidson SE, Davies NW, Smith JA, Dalmais M, Bendahmane AI, Quittenden LJ, Sutton L, Bala RK, Le Signor C, Thompson R, Horne J, Reid JB, Ross JJ (2012) Biosynthesis of the halogenated auxin, 4-chloroindole-3-acetic acid. Plant Physiol 159:1055–1063. 10.1104/pp.112.198457 22573801 PMC3387693

[CR116] Tivendale ND, Ross JJ, Cohen JD (2014) The shifting paradigms of auxin biosynthesis. Trends Plant Sci 19:44–51. 10.1016/j.tplants.2013.09.012 24524164

[CR117] Tsurumi S, Wada S (1986) Dioxindole-3-acetic acid conjugates formation from indole-3-acetylaspartic acid in *Vicia* seedlings. Plant Cell Physiol 27:1513–1522. 10.1093/oxfordjournals.pcp.a077252

[CR118] Včelařová L, Skalický V, Chamrád I, Lenobel R, Kubeš MF, Pěnčík A, Novák O (2021) Auxin metabolome profiling in the *Arabidopsis* endoplasmic reticulum using an optimised organelle isolation protocol. Int J Mol Sci 22:9370. 10.3390/ijms22179370 34502279 PMC8431077

[CR119] Wang B, Chu J, Yu T, Xu Q, Sun X, Yuan J, Xiong G, Wang G, Wang Y, Li J (2015) Tryptophan-independent auxin biosynthesis contributes to early embryogenesis in *Arabidopsis*. Proc Natl Acad Sci U S A 112:4821–4826. 10.1073/pnas.1503998112 25831515 PMC4403211

[CR120] Williams WR (2021) Phytohormones: structural and functional relationship to purine nucleotides and some pharmacologic agents. Plant Signal Behav 16:1837544. 10.1080/15592324.2020.1837544 33100143 PMC7781725

[CR121] Wright M, Dawson J, Dunder E, Suttie J, Reed J, Kramer C, Chang Y, Novitzky R, Wang H, Artim-Moore L (2001) Efficient biolistic transformation of maize (*Zea mays* L.) and wheat (*Triticum aestivum* L.) using the phosphomannose isomerase gene, *pmi*, as the selectable marker. Plant Cell Rep 20:42–436. 10.1007/s002990100318 24549451

[CR122] Xu T, Dai N, Chen J, Nagawa S, Cao M, Li H, Zhou Z, Chen X, De Rycke R, Rakusová H, Wang W, Jones AM, Friml J, Patterson SE, Bleecker AB, Yang Z (2014) Cell surface ABP1-TMK auxin-sensing complex activates ROP GTPase signaling. Science 28:1025–1028. 10.1126/science.1245125 PMC4166562 24578577

[CR123] Yu Y, Tang W, Lin W, Li W, Zhou X, Li Y, Chen R, Zheng R, Qin G, Cao W, Pérez-Henríquez P, Huang R, Ma J, Qiu Q, Xu Z, Zou A, Lin J, Jiang L, Xu T, Yang Z (2023) ABLs and TMKs are co-receptors for extracellular auxin. Cell 186:5457-5471.e17. 10.1016/j.cell.2023.10.017 37979582 PMC10827329

[CR124] Zhang J, Lin JE, Harris C, Pereira FCM, Wu F, Blakeslee JJ, Peer WA (2016) DAO1 catalyzes temporal and tissue-specific oxidative inactivation of auxin in *Arabidopsis thaliana*. Proc Natl Acad Sci U S A 113:11010–11015. 10.1073/pnas.1604769113 27651492 PMC5047167

[CR125] Zhao Z, Zhang Y, Liu X, Zhang X, Liu S, Yu X, Ren Y, Zheng X, Zhou K, Jiang L, Guo X, Gai Y, Wu C, Zhai H, Wang H, Wan J (2013) A role for a dioxygenase in auxin metabolism and reproductive development in rice. Dev Cell 27:113–122. 10.1016/j.devcel.2013.09.005 24094741

[CR126] Zheng Z, Guo Y, Novák O, Dai X, Zhao Y, Ljung K, Noel JP, Chory J (2013) Coordination of auxin and ethylene biosynthesis by the aminotransferase VAS1. Nat Chem Biol 9:244–246. 10.1038/nchembio.1178 23377040 PMC3948326

[CR127] Zolman BK, Nyberg M, Bartel B (2007) IBR3, a novel peroxisomal acyl-CoA dehydrogenase-like protein required for indole-3-butyric acid response. Plant Mol Biol 64:59–72. 10.1007/s11103-007-9134-2 17277896

[CR128] Zolman BK, Martinez N, Millius A, Adham AR, Bartel B (2008) Identification and characterization of *Arabidopsis* indole-3-butyric acid response mutants defective in novel peroxisomal enzymes. Genetics 180:237–251. 10.1534/genetics.108.090399 18725356 PMC2535678

